# Whole Genome Sequencing and Complete Genetic Analysis Reveals Novel Pathways to Glycopeptide Resistance in *Staphylococcus aureus*

**DOI:** 10.1371/journal.pone.0021577

**Published:** 2011-06-27

**Authors:** Adriana Renzoni, Diego O. Andrey, Ambre Jousselin, Christine Barras, Antoinette Monod, Pierre Vaudaux, Daniel Lew, William L. Kelley

**Affiliations:** Service of Infectious Diseases, Geneva University Hospitals, Geneva, Switzerland; University of Liverpool, United Kingdom

## Abstract

The precise mechanisms leading to the emergence of low-level glycopeptide resistance in *Staphylococcus aureus* are poorly understood. In this study, we used whole genome deep sequencing to detect differences between two isogenic strains: a parental strain and a stable derivative selected stepwise for survival on 4 µg/ml teicoplanin, but which grows at higher drug concentrations (MIC 8 µg/ml). We uncovered only three single nucleotide changes in the selected strain. Nonsense mutations occurred in *stp1*, encoding a serine/threonine phosphatase, and in *yjbH*, encoding a post-transcriptional negative regulator of the redox/thiol stress sensor and global transcriptional regulator, Spx. A missense mutation (G45R) occurred in the histidine kinase sensor of cell wall stress, VraS. Using genetic methods, all single, pairwise combinations, and a fully reconstructed triple mutant were evaluated for their contribution to low-level glycopeptide resistance. We found a synergistic cooperation between dual phospho-signalling systems and a subtle contribution from YjbH, suggesting the activation of oxidative stress defences via Spx. To our knowledge, this is the first genetic demonstration of multiple sensor and stress pathways contributing simultaneously to glycopeptide resistance development. The multifactorial nature of glycopeptide resistance in this strain suggests a complex reprogramming of cell physiology to survive in the face of drug challenge.

## Introduction

Methicillin-resistant *Staphylococcus aureus* (MRSA) infections is a major cause of morbidity and mortality worldwide, including in the health care setting. The mortality rate associated with invasive MRSA infections is significant and in the United States MRSA infections are now quoted as the leading cause of death by an infectious agent [1]. MRSA infections are particularly difficult to treat because of the development of antibiotic resistance and limited therapeutic options. Glycopeptide antibiotics (vancomycin and teicoplanin) are still the preferred drugs for treatment of serious hospital or community-acquired MRSA infections, despite reports of increasing numbers of glycopeptide-resistant MRSA isolates [2], [3].

Glycopeptide resistance in *Staphylococcus* have emerged by two mechanisms. Highly glycopeptide-resistant *S. aureus* strains (VRSA; MIC≥16 µg/ml) acquired the exogeneous multigene VanA complex carried on transposon Tn*1546* from *Enterococcus faecalis* by horizontal gene transfer. Fortunately, these events are infrequent and only few examples are known worldwide [4], [5]. The second mechanism of resistance, termed endogenous or low-level (MICs with >2 µg/ml to <16 µg/ml), arises because spontaneous mutation(s) are thought to confer a selective survival advantage. Endogeneous resistance is thought to occur stepwise: emergence of resistance to low-antibiotic levels must be first acquired to allow growth in progressively higher antibiotic concentrations [6], [7]. The exact molecular mechanism(s) leading to endogeneous resistance to teicoplanin, or vancomycin, is/are unknown. A common resistance pathway has being suggested since in general reduced susceptibility to vancomycin strains also display reduced teicoplanin sensitivity. However, teicoplanin resistance can be acquired without alteration in vancomycin susceptibility [3], [6].

Endogenous resistance is more often observed and clinical studies have linked glycopeptide clinical failure with progressive selection of bacterial isolates showing increasing glycopeptide resistance levels. In some reported cases, as little as a two-fold change in MIC altered clinical outcome [8], [9]. Such concerns have resulted in the recent re-evaluation of clinical breakpoints for glycopeptides [10] (www.srga.org/). In light of these concerns, understanding the molecular changes permitting survival of *S. aureus* during drug challenge is of paramount importance.

Glycopeptides are non-penetrating cell wall acting antibiotics whose site of action lies outside the cell membrane, implying that changes in physical-chemical barriers, detection, signalling and response mechanisms could conceivably promote resistance. Several phenotypic responses leading to resistance are observed in some, but not all cases and include thicker cell wall, reduced autolysis and increased cell wall crosslinking [11], [12], [13], [14], [15], [16], [17]. These changes are thought to be correlated with a differential expression of genes involved in cell wall metabolism. In several studies, point mutations occurring in only one gene (*vraRS*, *graRS* or *tcaA*) have been described and confirmed by genetic analysis to contribute to glycopeptide resistance [15], [18], [19]. No studies concerning the functional links between these pathways have been provided, however. Only one study has uncovered multiple mutations by sequence analysis when comparing Mu50 (VISA) and a revertant Mu50Ω (VSSA) that were conclusively proven to contribute to low-level glycopeptide resistance [20].

In this study, we identified genomic changes using whole genome deep sequencing of a teicoplanin-susceptible and its teicoplanin-resistant derivative. Point mutations occurred in three genes and complete genetic analysis proved their causal link to glycopeptide resistance. We demonstrate multiple pathways contributing to glycopeptide resistance emergence including two types of phosphosignalling (histidine and serine/threonine) combined with a global redox/thiol stress sensor.

## Results

### Genomic sequencing of isogenic ISP794 and AR376 strains

To identify genomic changes selected during teicoplanin exposure, whole genome deep sequencing of both ISP794 and AR376 was performed. AR376 is an in vitro derived stable teicoplanin-resistant mutant from laboratory strain ISP794 [21].

Since ISP794 and AR376 are derivatives of the sequenced NCTC8325 strain (NCBI Accession No. NC_007795), we used this as a reference to evaluate genome coverage and facilitate contig assembly. Using Illumina-Solexa technology, we obtained 5,659,670 and 3,609,916 of 35-bp paired-end reads for ISP794 and AR376 strains, respectively, mapping the reference genome. The raw coverage depth was 35 and 23 times for ISP794 and AR376, respectively. Mapping covered 2,819,664 and 2,819,622 bases in ISP794 and AR376 strains, resulting in 99.8% and 99.6% of NCTC8325 genome coverage (2,821,361 bp), respectively. Most of the reference genome was covered sufficiently (minimum coverage of 3 read bases per reference base) to allow single nucleotide polymorphism (SNP) detection. The paired end information was used to detect with high confidence insertions and deletions (InDel).

### SNP and InDel differences detected between ISP794 and AR376 strains

**Figure 1:**
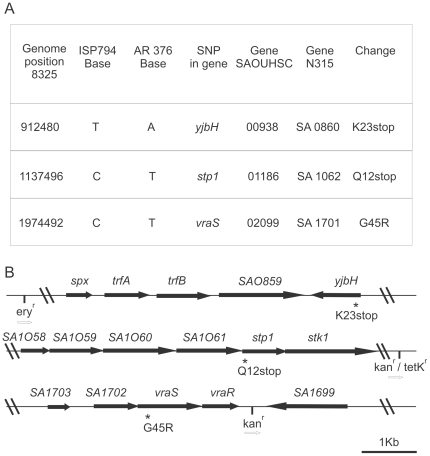
Genomic differences detected by whole genome sequencing. (A) Chromosomal location of detected SNPs and predicted protein changes. (B) Physical map of each locus containing *yjbH*(K23stop), *stp1*(Q12stop) and *vraS*(G45R). For genetic analysis, each mutation was tagged by site-specific insertion of a nearby selectable marker to facilitate strain constructions using bacteriophage-mediated transduction.

Computer analysis of interstrain differences between the assembled ISP794 and AR376 genomes first revealed 9 potential SNPs and 11 potential InDels. Each difference was subsequently re-examined by genomic DNA PCR amplification using appropriate primers and sequencing. Six potential SNPs and all InDel differences were rejected by this analysis. As shown in Figure 1, only 3 SNP differences apparently distinguished AR376 from its parental strain ISP794.

SNPs were detected in three different open reading frames corresponding to reference sequence tag SAOUHSC 00938, SAOUHSC 01186 and SAOUHSC 02099 genes (equivalent to N315 ordered sequence tags SA0860, SA1062 and SA1701, respectively). Similarity searches with SAOUHSC 00938 indicated the presence in *Bacillus subtilis* of a gene showing partial similarity and annotated as *yjbH* (SwissProt accession BG13137). In *B. subtilis* YjbH is an adaptor protein, which, together with ClpXP protease, regulates the degradation of the global transcriptional regulator Spx [22]. In *S. aureus*, YjbH is a hypothetical protein showing 34% identity and 73% amino acid sequence similarity with YjbH of *B. subtilis*. The *yjbH* t/a SNP occurred at nucleotide position 67 generating a nonsense mutation at amino acid 23 (K23stop). The SAOUHSC 01186 gene encodes Stp1, a serine/threonine phosphatase [23]. The *stp1* c/t SNP occurred at nucleotide position 34 generating a nonsense mutation at amino acid 12 (Q12stop). Finally, the SAOUHSC 02099 gene encodes the histidine kinase sensor VraS. The *vraS* c/t SNP occurred at nucleotide position 133 generating a missense mutation at amino acid 45 by substituting arginine for glycine (G45R) (Figure 1). The amino acid G45 in VraS is located in a region predicted to lie between two N-terminal transmembrane domains suggesting a possible role in extracellular signal sensing.

### Genetic analysis of each SNP and its contribution to teicoplanin-resistance

**Figure 2:**
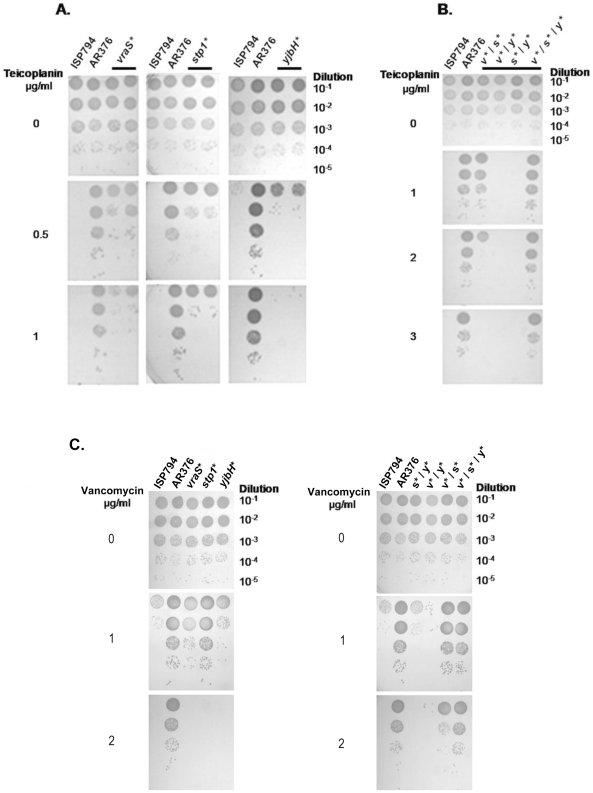
Stepwise genetic reconstitution of all SNP permutations and analysis of their contribution to glycopeptide resistance. (A and B) Teicoplanin spot plating population analysis (Spot PAP) on teicoplanin of ISP794 and each of its strain derivatives. Spot serial dilutions are indicated at the right margin. The first spot 10 µl corresponds to 1×10^5^ colony forming units (CFU). Results for two independent isolates are shown for each single mutant (solid bar). For convenience, genes are marked with an asterisk such that vraS*, stp1* and yjbH* correspond to *vraS*(G45R), *stp1*(Q12stop) and *yjbH*(K23stop) mutations, respectively. (C) Vancomycin spot plating population analysis (Spot PAP) of ISP794 and each of its strain derivatives. Conditions used were as in panels A, B.

To determine whether each individual SNP detected in AR376 contributed to reduced teicoplanin susceptibility, we first re-engineered each SNP change by site-specific mutation or phage mediated transduction backcross in ISP794 (see Table 1). As judged by broth macrodilution MICs and confirmed by spot population analysis profiles (spot PAP assays), *vraS*(G45R), or *stp1*(Q12stop), led to reduced teicoplanin susceptibility (MIC = 2 µg/ml) compared to the parent ISP794 (MIC = 1 µg/ml) (Table 2). Spot PAP assays revealed detectably enhanced growth of both single mutants on MHA supplemented with increasing concentrations of teicoplanin, while no growth was observed with parent ISP794. Identical results were obtained when comparing a second independent isolate of each single mutant (Figure 2A). In contrast to *vraS*(G45R) and *stp1*(Q12stop) mutants, a small increase of growth was observed for *yjbH*(K23stop) while no growth was observed for the parent ISP794. Growth was observed when 1×10^5^ and 1×10^4^ colony forming units were applied on MHA supplemented with 0.5 µg/ml of teicoplanin (Figure 2A). However, MIC analysis showed no difference between ISP794 and its isogenic *yjbH*(K23stop) mutant (MIC = 1 µg/ml) (Table 2).

**Table 1:** Strains and plasmids used in this study.

Strain/plasmid	Revelant genotype	Characteristics	Source/reference
***S. aureus***			
RN4220	8325-4; r- m+, restriction defective laboratory strain		[55]
ISP794	8325 *pig* 131		[56]
AR376	ISP4-2-1		[21]
AR774	*vraS* (G45R)	ISP794, *vraS* (G45R) kan^r^ nearby	This study
AR758	kan^r^ nearby *vraS*	ISP794, *vraS* kan^r^ nearby	[32]
AR756	*vraS*::*ermB*	ISP794, *vraS::ermB*	[32]
AR802	kan^r^ nearby *stp1*(Q12stop)	AR376, *stp1*(Q12stop) kan^r^ nearby	This study
AR860	kan^r^ nearby *stp1*	ISP794, *stp1*kan^r^ nearby	This study
AR826	*stp1* (Q12stop)	ISP794, *stp1*(Q12stop) kan^r^ nearby	This study
AR853	tetK^r^ nearby *stp1*(Q12stop)	AR376, *stp1*(Q12stop) tetK^r^ nearby	This study
AR858	*vraS* (G45R), *stp1* (Q12stop)	ISP794, vraS (G45R) kan^r^ nearby, *stp1*(Q12stop) tetK^r^ nearby	This study
AR864	*vraS* (G45R), *yjbH* (K23stop)	AR376, *stp1* kan^r^ nearby	This study
AR854	*stp1 (Q12stop)*, *yjbH* (K23stop)	AR376, *vraS* kan^r^ nearby	This study
AR1077	ery^r^ nearby *yjbH* (K23stop)	AR376, *yjbH* (K23stop) ery^r^ nearby	This study
AR1079	*yjbH* (K23stop)	ISP794, yjbH(K23stop) ery^r^ nearby	This study
AR1082	*vraS* (G45R), *yjbH* (K23stop), *stp1* (Q12stop)	ISP794, *vraS* (G45R) kan^r^ nearby, *yjbH* (K23stop) ery^r^ nearby, *stp1*(Q12stop) tetK^r^ nearby	This study
AR964	AR774/pAM1483		This study
AR1001	AR826/pAR992		This study
AR1085	AR1082/pAR973		This study
SaΔ*clpP*	8325-4 derived strain	*clpP* deleted strain	[57]
Δspx	8325-4 derived strain	*spx* deleted strain	[27]
spx^+^	8325-4 Δspx, P_spx_-spx::geh	*spx* deleted strain and chromosomally complemented with the intact copy of *spx* inserted into *geh* locus.	[27]
***Plasmids***			
pTYB12	N-terminal fusion IMPACT intein and chitin binding domain plasmid		New England (Biolabs)
pMK4	*E.coli*-*S.aureus* shuttle vector, amp^r^ and cam^r^		[58]
pBT2	*E.coli*-*S.aureus* thermosensitive-shuttle vector, amp^r^ and cam^r^		[59]
pBluescript II KS+	routine multicopy *E.coli* cloning vector amp^r^		Stratagene
pAR749	pBT2, *vraS-*G45R kan^r^ nearby ts shuttle vector		This study
pAR712	pBT2, *vraS*-kan^r^-SA1699 intergenic ts shuttle vector		[32]
pAR784	pBT2, *stp1*-kan^r^ nearby ts shuttle vector		This study
pAR787	pBT2, *stp1*-tet^r^ nearby ts shuttle vector		This study
pAR1063	pBT2, *yjbH*-ery^r^ nearby ts shuttle vector		This study
pAM1483	pMK4- 3.3 kb entire *vraR* operon and upstream promoter region Kpn-Pst		32
pAR992	pMK4- containing Not1-Kpn pHU promoter region, Kpn-Pst1 *Stp1* gene		This study
pAR973	pMK4- containing Not1-Kpn pGlyS promoter region, Kpn-Pst1 *yjbH* gene		This study
pAM1101	pTYB12-SpxA (Nde-Pst)		This study

**Table 2:** Glycopeptide susceptibility profiles of ISP794 and its derivatives.

	MIC* µg/ml	
Strain	Teicoplanin	Vancomycin
ISP794	1	2
AR376	8	4
*vraS* ^*^	2	2
*stp1* ^*^	2	2–4
*yjbH* ^*^	1	2
*vraS* ^*^ */stp1* ^*^	4	4
*vraS* ^*^ */yjbH* ^*^	1	2
*stp1* ^*^ */yjbH* ^*^	1	2
*vraS* ^*^ */stp1* ^*^ */yjbH* ^*^	8	4

*MIC, Modal minimum inhibitory concentration measured by broth macrodilution. VraS^*^, stp1^*^ and *yjbH* ^*^ correspond to *vraS* (G45R), *stp1* (Q12stop) and *yjbH* (K23stop) mutations, respectively.

Complementation of either *vraS*(G45R) or *stp1*(Q12stop) mutants by a multicopy plasmid carrying the corresponding wild-type genes, led to restored sensitivity to teicoplanin (Figure S1). As *yjbH*(K23stop) mutation has a subtle effect on teicoplanin resistance, complementation of the *yjbH* mutation was performed by introducing a multicopy plasmid carrying *yjbH* wild-type gene into the triple mutant (see below), a condition where its role could be readily assessed. We observed restored sensitivity of the triple mutant to levels comparable with the double mutant *vraS*(G45R)/*stp1*(Q12stop) mutant (Figure S1).

Collectively, we conclude that single *vraS* (G45R), *stp1* (Q12stop) and, to a lesser extent, *yjbH*(K23stop) mutations in parent ISP794 decreased teicoplanin susceptibility, however, none of the single mutations alone could account for the parental AR376 MIC levels. We conclude that some combination of these mutants must therefore contribute to the observed teicoplanin-resistant phenotype of AR376.

### Effect of mutant combinations on teicoplanin resistance

We next analysed the drug resistance phenotypes of each of the three possible pairwise mutants: *stp1/yjbH*, *stp1/vraS*, and *yjbH/vraS*. As shown in Figure 2B and Table 2, a further decrease of teicoplanin susceptibility was observed only with double mutant *vraS*(G45R)-*stp1*(Q12stop). Spot PAP assays revealed growth of the double *vraS*(G45R)-*stp1*(Q12stop) mutant on MHA supplemented with 2 µg/ml teicoplanin and showing a macrodilution MIC of 4 µg/ml. Notably, the double mutant displayed enhanced growth compared to either of the single mutants alone (Figure 2A and B, compare growth and log_10_ –fold scale, teicoplanin 1 µg/ml panels, for the single *stp1* or *vraS* mutants *versus* the *stp1/vraS* double mutant). In contrast, and as detected by spot PAP assay and macrodilution MIC, the other two pairwise mutants did not show any decrease in teicoplanin susceptibility (Figure 2B and Table 2). Interestingly, the decrease in teicoplanin susceptibility observed in *vraS*(G45R), or *stp1*(Q12stop) single mutants, was abolished by the *yjbH*(K23stop) mutation (Figure 2A, B and Table 2). These results suggest that the loss of *yjbH* exerts a negative effect on the pathways leading to drug sensitivity changes engendered by *vraS*(G45R) or *stp1*(Q12stop) mutations.

Although the double mutant *vraS*(G45R)-*stp1*(Q12stop) decreased teicoplanin susceptibility, it still did not fully recapitulate the AR376 MIC levels. The effect on teicoplanin susceptibility of the fully reconstructed triple mutant was therefore tested. Genetic analysis of the re-engineered triple mutant revealed complete restoration of the teicoplanin resistance phenotype indistinguishable from AR376. Spot PAP assays revealed identical growth of both the triple mutant *vraS*(G45R) - *stp1*(Q12stop) - *yjbH*(K23stop) and AR376 on MHA supplemented with increasing concentrations of teicoplanin (Figure 2B) and identical macrodilution MIC levels were also observed (MIC = 8 µg/ml) (Table 2).

### Effect of single mutations and their combinations on vancomycin susceptibility

The impact of all mutations was also examined for their contribution to changes in vancomycin susceptibility, despite the fact that the *in*-*vitro* derived teicoplanin-resistant AR376 strain showed only a 2-fold marginal increase in vancomycin MIC compared to its susceptible counterpart ISP794 (Table 2). Changes in vancomycin MICs were not as dramatic as the changes observed in teicoplanin MICs for the various mutant combinations (Table 2). Nevertheless, as shown in Figure 2C, similar results with teicoplanin susceptibilities were observed using the more sensitive log_10_-scaled growth assay. Spot PAP assays revealed detectable enhanced growth of both single *vraS*(G45R) or *stp1*(Q12stop) mutants on MHA supplemented with 1 µg/ml of vancomycin compared to the susceptible strain ISP794. In contrast no difference was observed with *yjbH*(K23stop) mutant. At 2 µg/ml vancomycin concentration only AR376 showed growth and comparable colony formation as observed with control agar plates with no drug. Further analysis of all pairwise and triple mutants revealed a detectable decrease in vancomycin susceptibility only for the double *vraS*(G45R)-*stp1*(Q12stop) and reconstructed triple mutants. As previously noted above and in results presented in Figure 2B, we also observed that the *yjbH*(K23stop) mutation, when paired with either *vraS*(G45R) or *stp1*(K12stop) exerted a negative effect by reversing drug sensitivity changes arising from these single mutations.

Taken together, we conclude that all three SNPs were collectively responsible for the decreased glycopeptide susceptibility of AR376 strain. Furthermore, *vraS*(G45R) and *stp1*(Q12stop) mutations acted synergistically to reduce glycopeptide susceptibility while *yjbH*(K23Stop) contributed significantly to low level glycopeptide resistance in this strain only when paired with the dual phospho-signalling mutants. These results also reveal that although AR376 was originally selected for reduced susceptibility to teicoplanin, the genetic changes also impact vancomycin susceptibility, *albeit* to a lesser degree in this strain background. The sensitivity of the spot PAP assay to reveal these subtle changes underscores its powerful application to unravelling the genetics of endogenous glycopeptide resistance.

### Analysis of cell wall thickness by electron microscopy

**Figure 3:**
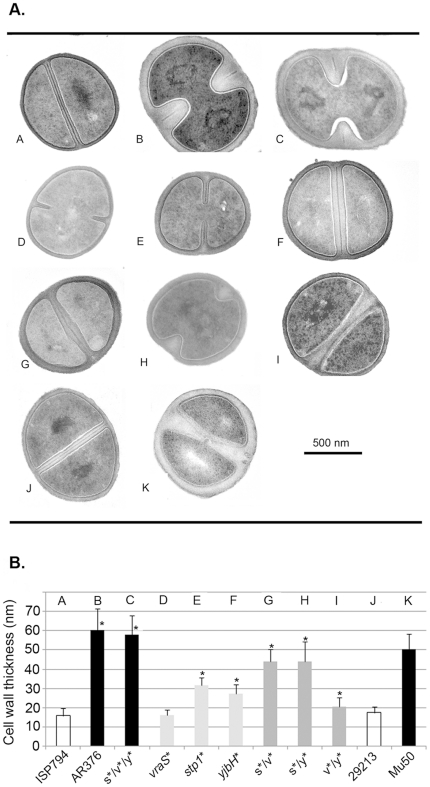
Cell wall thickness analysis. (A) Transmission electron microscopy showing one representative image of each bacterial strain used in this study, after growth in the absence of antibiotic to post-exponential phase on TSB media. Magnification ×37,000. Scale bar automatically inserted by the microscope imaging system is shown. (B) Quantification of cell wall thickness (nm) of each bacterial strain. Reported values correspond to the mean ± SD (n = at least 40) of each bacterial strain. Asterisk (*) represents results significantly different by student's two-tailed t-test (*p*<0.05) from ISP794.

Since the VraRS two-component sensor system regulates a response to cell wall stress and controls, in part, the expression of genes involved cell wall metabolism [24], and further, since *stk1*/*stp1* modulate cell wall metabolism [25], we analysed the cell wall thickness of all strains used in this study by transmission electron microscopy (Figure 3). As reference control strains, we first analysed the cell wall thickness of the glycopeptide resistant strain Mu50, which displays a significantly thicker cell wall (50.4±8 nm) than the glycopeptide susceptible control strain ATCC29213 (17.35±2.9 nm) (Figure 3A and 3B; [11]). Analysis of AR376 showed a significantly (*p*<0.001) thicker cell wall compared to its susceptible counterpart ISP794 and furthermore, this enhanced thickness was comparable to, and in fact greater than, Mu50. Both of the *stp1*(Q12stop) and *yjbH*(K23stop) single mutations significantly increased (*p*<0.001) cell wall thickness compared to the susceptible parental strains ISP794, while in contrast, no significant difference was observed between *vraS*(G45R) mutant and ISP794. Analysis of all pairwise mutants showed a significantly greater (*p*<0.001) increase in cell wall thickness compared to ISP794; however, no double mutation attained AR376 cell wall thickness levels. Indeed, the observed mean thickness for both the *stp1/vraS* and *stp1/yjbH* mutations were significantly less (p<0.001) than AR376. Only the fully reconstituted triple mutant AR1082 showed comparable cell wall thickness as AR376 strain. It is worthwhile noting that the *yjbH*(K23stop) mutation enhanced cell wall thickness when paired with either *stp1*(Q12stop) or *vraS*G45R in these assays in contrast to the negative effect of *yjbH*(K23stop) on teicoplanin MIC and spot PAP assay growth (on either teicoplain or vancomycin) when paired with these mutants as described above. Apart from these changes in cell wall thickness, we did not observe overt changes in cell division such as aberrant septum formation or other peculiar morphology for any mutation examined.

Collectively, these results show that all mutations contribute to significant alterations in cell wall thickness in our strain background; however, the observed changes in cell wall thickness do not precisely correlate with the observed changes in glycopeptide susceptibility. We conclude that changes in glycopeptide sensitivities in this strain background must arise primarily from physiological changes other than pronounced changes in cell wall thickness.

### YjbH negatively regulates Spx levels in *S. aureus*

A role for YjbH has not been previously described in *S. aureus*; however, in *B. subtilis*, YjbH acts as a post-transcriptional negative regulator of the global oxidative/thiol stress regulator Spx [26]. YjbH binds Spx and functions as an adaptor protein directing ClpXP-dependent degradation of Spx. Disruption of *yjbH* or *clpP* in *B. subtilis* is known to result in enhanced levels of Spx since its proteolytic turnover is greatly attenuated. A rise in Spx levels leads to substantial changes in gene expression profiles.

**Figure 4:**
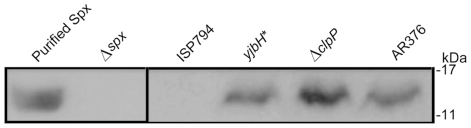
Western blot showing the effect of *yjbH*(K23stop) mutation on Spx protein levels. Total soluble protein extracts (50 µg) from *S. aureus* strains were loaded per well in an SDS 15% acrylamide gel. Spx protein (15 kDa) was detected using rabbit-polyclonal anti-Spx antibodies. Purified-Spx protein (600 ng) (lane 1), Δ*spx* strain (lane 2), ISP794 containing wild-type *yjbH* gene (lane 3), yjbH* correspond to *yjbH(K23stop)* mutant (lane 4), SaΔ*clpP* strain (lane 5) and AR376 (lane 6). The position of protein markers are shown in the right margin.

To address the hypothesis that *S. aureus* YjbH acts as a negative regulator of Spx analogous to its *B. subtilis* counterpart, we first examined Spx protein levels by western blot. As shown in Figure 4, rabbit polyclonal anti-Spx antibody recognized purified *S. aureus* Spx migrating with the expected molecular weight of 15 kDa. No Spx was detected in a negative control *S. aureus* 8325-4 derived strain extract containing an internal disruption of *spx*, whereas Spx was strongly detected in cell extract lacking *clpP* as previously described [27]. Spx was also detected in an extract from AR376, harbouring the triple *vraS*(G45R)-*stp1*(Q12stop)-*yjbH*(K23stop) mutant combination. Importantly, comparison of extracts from ISP794 and its isogenic derivative AR1079 containing the *yjbH*(K23Stop) mutation, showed that Spx was undetectable in the presence of wild type *yjbH*, but clearly detectable in the presence of the *yjbH*(K23Stop). We conclude from these results that the loss of *yjbH* results in significant stabilization of Spx protein in *S. aureus* and that YjbH probably functions similarly to its *B. subtilis* counterpart by aiding Spx proteolytic turnover. Consequently, the triple mutant AR376 harbors changes in two distinct phosphosignalling pathways and possesses enhanced Spx protein levels arising from the loss of its negative regulator *yjbH*.

### Influence of Spx and teicoplanin on transcription of *msrA1* encoding methionine sulfoxide reductase in *S. aureus*

Several lines of evidence link oxidative stress and *S. aureus* responses to glycopeptide antibiotics. First, recent work suggests that the mode of killing by several classes of antibiotic, including glycopeptides, involves the endogenous production of reactive oxygen radicals [28]. Secondly, transcriptional profiling revealed that an oxidative stress defence gene, *msrA1*, encoding methionine sulfoxide reductase was among those genes most strongly upregulated in response to various cell wall active antibiotics, including glycopeptides, in *S. aureus* [29], [30]. As *msrA1* transcription is known to be positively regulated by Spx in *B. subtilis* [31], we hypothesized that a possible functional consequence of the loss of *yjbH* in AR376, would be enhanced transcription of Spx-dependent genes such as *msrA1* (N315 ordered sequence tag SA1257). To explore this possibility, we tested whether *msrA1* was indeed subject to Spx-dependent transcription regulation in *S. aureus*.

**Figure 5:**
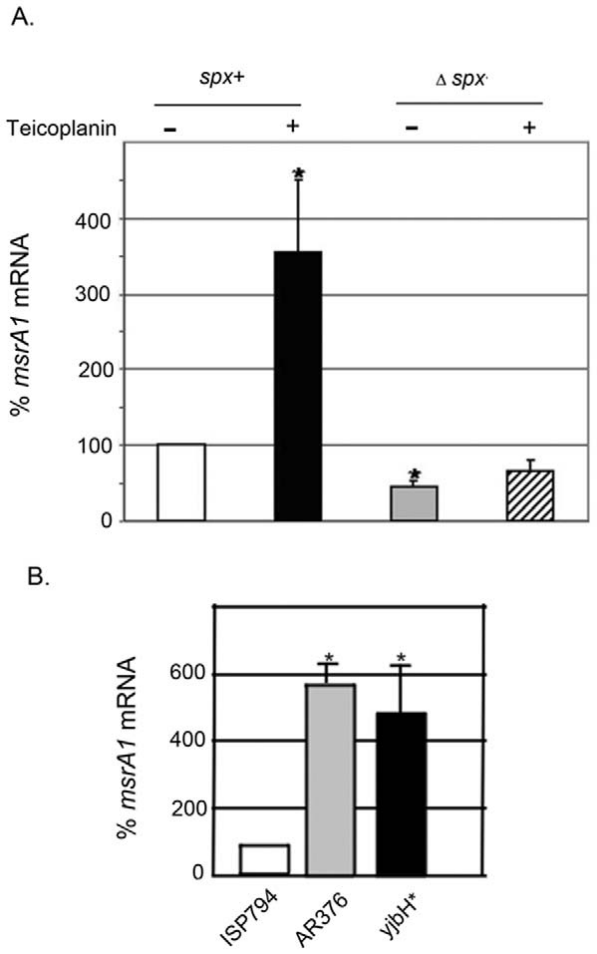
Effect of *spx* on *msrA1* transcription. (A) Steady-state levels of *msrA1* transcript of wild type strain (*spx* ^+^) compared to mutant Δ*spx* strain, determined by qRT-PCR. Values represent the mean ± SEM of four independent experiments performed in triplicate. Data are depicted setting *spx* ^+^ values in the absence of teicoplanin as 100%. Asterisk (*) represents results significantly different by student's two-tailed t-test (*p*<0.05) from *spx* ^+^ without teicoplanin. (B) Steady-state levels of *msrA1* transcript of ISP794 containing wild-type *yjbH* gene, AR376 and yjbH* containing both *yjbH(K23stop)* mutation, determined by qRT-PCR. Values represent the mean ± SEM of three independent experiments performed in triplicate. Data are depicted setting ISP794 values as 100%. Asterisk (*) represents results significantly different by student's two-tailed t-test (*p*<0.05) from ISP794.

The results (Figure 5A) show that in a Δ*spx* strain, teicoplanin exposure resulted in no significant induction of *msrA1* compared to uninduced control, whereas in the Δ*spx* strain complemented with a chromosomally integrated copy of wild type *spx*, *msrA1* induction was significantly induced more than 3-fold (*p*<0.05) following exposure to teicoplanin. Indeed, in the absence of *spx*, even basal *msrA1* transcript levels were significantly lower (*p*<0.05) compared with *msrA1* transcript levels in the *spx* complemented strain.

Since the *yjbH*(K23stop) mutation results in significant stabilization of Spx protein, we also analysed whether the steady state *msrA1* transcription in the absence of drug induction was affected in either AR376 or the *yjbH*(K23stop) single mutant. Figure 5B shows that basal *msrA1* transcript levels were indeed significantly higher (*p*<0.05) in both AR376 and *yjbH*(K23stop) mutant compared to the parental strain ISP794. We conclude from these results that *msrA1* is subject to transcriptional regulation dependent upon *spx* in *S. aureus*.

We also recently described the discovery of *trfA* (teicoplanin resistance factor A) as a gene whose loss restores glycopeptide sensitivity in AR376 [21]. We have found that *trfA* transcript levels are also significantly increased by the loss of *yjbH* and that the *trfA* promoter is also positively regulated by Spx (A. Renzoni, manuscript in preparation). Taken together, these findings strongly suggest that stabilization of Spx through disruption of *yjbH* results in important functional consequences within the cell since Spx positively regulates the expression of at least two genes, *msrA1* and *trfA*, known to contribute to glycopeptide resistance.

### Effect of pre-existing *stp1*(Q12stop), *yjbH*(K23stop), *vraS*(G45R) or *spx* mutations on the emergence teicoplanin resistance in *S. aureus*

Since AR376 was originally obtained by stepwise cultivation on agar plates containing low levels of teicoplanin we wished to determine the extent to which each individual mutation identified in our study contributed to a change in a detectable frequency of emergence of teicoplanin resistant colonies using our standard assay conditions [21], [32]. In light of our findings that disruption of *yjbH* altered Spx levels, we also tested the effect of *spx* deletion in the same assay. The results are shown in Table 3.

**Table 3:** Effect of the single point mutations and *spx* on the emergence of teicoplanin resistance.

		No. bacteria under non-selective conditions T_0_		No. bacteria under selective conditions T_2_		Frequency of emergence^1^	
Strain	Relevant genotype	Mean (n = 7)	SEM	Mean (n = 7)	SEM	T_2_/T_0_	%
ISP794	ISP794, *rsbU* ^−^	7.82×10^+7^	2.01×10^+7^	255	79	3.26×10^−6^	100^3^
AR1079	ISP794, *yjbH(K23stop)* ery^r^ nearby	6.98×10^+7^	1.95×10^+7^	895	201	1.28×10^−5^	394
AR774	ISP794, *vraS(G45R) kan* ^r^ nearby	1.25×10^+7^	2.24×10^+6^	>1000^2^		8.00×10^−5^	>1000
AR826	ISP794, *stp1(Q12stop)* kan^r^ nearby	2.80×10^+7^	5.73×10^+6^	>1000^2^		3.50×10^−5^	>1000

		Mean (n = 5)	SEM	Mean (n = 5)	SEM		
*spx*+	8325-4Δ*spx, P_spx_-spx::geh*	1.59×10^+8^	1.61×10^+7^	>1000^2^		>6.00×10^−6^	100^4^
Δ*spx*	8325-4Δ*spx*	1.05×10^+8^	1.39×10^+7^	49	12	4.70×10^−7^	<10

1 Frequency of emergence expressed as the ratio of colony forming units (CFU) under selective (Teicoplanin 2 µg/ml) and non-selective conditions (No teicoplanin). Colony forming units were counted at 48 h, 37°C.

2 Viable counts on agar containing 2 µg/ml of teicoplanin were too high to accurately measure using these conditions. More than 1000 CFU were estimated in each experiment.

3 The effect of *rsbU* ^+^ on glycopeptide emergence is discussed in Galbusera et al [(30)]. ISP794 emergence frequency was set equal to 100% for normalization and comparison.

4 The emergence frequency was set equal to 100% for normalization and comparison with its derivative Δ*spx*.

Compared to ISP794, strains harboring any of the three mutations, *stp1*(Q12Stop), *yjbH*(K23Stop) or *vraS*(G45R) - each backcrossed by bacteriophage mediated transduction into ISP794 and never having been previously exposed to glycopeptides, resulted in strongly enhanced frequency (from 4- to 10-fold) of emergence of low-level teicoplanin resistant colonies on MH agar plates. In contrast, loss of *spx* was associated with at least a 10-fold reduction in the frequency of emergence of teicoplanin resistant colonies compared to the identical strain background harboring a second wild-type copy of *spx*. We conclude that loss of *spx* reduced the emergence of teicoplanin resistant colonies while mutation in any of the three mutations discovered by genomic sequence analysis of AR376 enhanced the emergence of teicoplanin resistance.

## Discussion

In the present study, we show by deep sequencing and genetic analysis that a laboratory selected low level teicoplanin intermediate resistant *S. aureus* strain has only three point mutations which distinguished it from its parental susceptible strain. These changes occur in two distinct (histidine kinase and serine/threonine kinase) phospho-signalling systems and in a gene contributing to a global oxidative/thiol stress defence response pathway. Moreover, we present evidence revealing a synergistic interaction between the dual phospho signalling pathways. The contribution of the third mutation in *yjbH* is subtle, and its properties suggest that it most probably exerts its effect following the first step acquisition of low level glycopeptide resistance mediated by the combined effects of *stp/vraS* mutations.

*S. aureus* glycopeptide resistance has been often correlated with morphological changes such as increased cell wall thickness, peptidoglycan crosslinking or decreased autolysis. Both *vraS* and *stp1* are known to affect cell wall metabolism, although precisely how is unknown [23], [24], [33], [34], [35].

The VraRS two-component system (TCS) responds to cell-wall active antibiotics [24], [33]. Under cell wall stress, expression of *vraRS* and several cell wall biosynthetic genes are strongly upregulated. Disruption of the histidine kinase *vraS* gene blocks this transcriptional response and reduces ß-lactam or glycopeptide resistance levels [32], [33], [36] Missense mutations in *vraS* altering the susceptibility to glycopeptides are commonly observed [17], [20], [37], [38].

How *vraS*(G45R) contributes to enhanced glycopeptide resistance is unknown. Amino acid 45 is located in the short region predicted to span two putative transmembrane segments and suggests a role in signal detection. The G45R mutation could also conceivably affect interaction with other regulatory proteins. For example, *B. subtilis* LiaF, a membrane protein, is thought to negatively regulate LiaS, a putative *S. aureus* VraS ortholog. *B. subtilis* LiaF is syngenic to *S. aureus* SA1702, and mutations in SA1702 have also been reported to affect glycopeptide resistance [17], [38]. Indeed, a recent report suggests that *S. aureus* SA1702/YvqF and VraS directly interact lending support to the notion that SA1702/YvqF may modulate VraS signalling and detection of cell wall stress [39].

The *vraS*(G45R) mutation does not detectably increase VraR operon mRNA levels compared to its isogenic parent strain, or any gene (*sgtB, prsA, htrA1, murZ*) thought to be regulated by VraR that we tested by qRT-PCR and our experimental conditions. Nevertheless, this mutation by itself does result in pronounced changes in emergence of glycopeptide resistance frequencies. This result is curious, since in most reported cases, glycopeptide resistant bacteria show increased expression of *vraR* operon transcription compared to susceptible parental strains [24], [37], [40]. Nevertheless, one report, consistent with our findings, showed that increased transcription of *vraS* or *vraRS* operon is not essential for acquisition of low-level glycopeptide resistance [41]. A closer study of *vraR* operon regulation and VraR phosphorylation mechanisms is warranted to resolve precisely how VraS missense mutations disrupt signalling.

In *S. aureus*, Stp1 plays a role in phospho-signalling, together with Stk1 [23], [34], [35], [42], [43]. *Stp1* (N315-SA1062), encodes a manganese-dependent serine/threonine phosphatase, capable of dephosphorylating Stk1 [23], [34]. Stk1 is thought to be a membrane protein which possesses three extracellular PASTA domains (penicillin-binding protein and serine/threonine kinase associated domain) [44]. Stk1 or Stp1 have been shown to have a role in *S. aureus* virulence, cell wall metabolism and antibiotic resistance (for review see [25]). The effects of a single *stp1* mutant, however, have not been tested for glycopeptide resistance [23], [34], [35], [42], [43]. To our knowledge, our work is the first study to report mutation in *stp1* alone directly affecting glycopeptide resistance levels.

It is not known how *stk1/stp1*-mediated signalling contributes to the detection of antibiotic encounter and orchestration of cellular responses. One plausible hypothesis is that Stk1 senses some feature(s) of the cell wall assembly process via its PASTA domains and phosphorylates multiple downstream factors that coordinate proper assembly and quality control [44]. In the event of encounter with cell wall active drugs such as glycopeptides, Stk1 could conceivably signal damage and initiate a cascade of signalling events that permit survival in the face of drug stress. The loss of function of *stp1* would have the predictable consequence of enhancing the duration of substrates phosphorylated by Stk1. Stk1 and Stp1 are only beginning to be understood in *S. aureus*, however. To date, several Stp1 client substrates have been described including the global accessory regulator SarA, MgrA, and the nucleoid organizing protein HU [43], [45]. MgrA is known to be a major cell-wall autolytic regulator which modulates antibiotic resistance, including glycopeptides [45], providing a possible mechanistic link between signal systems and altered cell wall assembly.

Changes in cell wall thickening resulting from *stp1* disruption appear to be strain dependent and divergent results have been reported for strains Newman and N315, for example [23], [34], [43]. The reason for these discrepancies is unknown and additional studies will be needed to resolve this important issue. The *stp1*(Q12stop) mutation present in our strain background nearly doubled cell wall thickness compared to ISP794. A recent metabolome study using 8325, a strain closely related to ISP794, detected striking coordinate increases in peptidoglycan intermediates depending upon the sequential action of MurA to MurF enzymes in a strain lacking *stp1* compared to its isogenic parent [46]. These results strongly suggest that *stk1/stp* are indeed intimately involved in the regulation of cell wall biosynthesis.

A striking feature of our study is the enhanced glycopeptide resistance of the double *stp1*(Q12stop)/*vraS*(G45R) mutation compared to the resistance levels of the single mutants alone. This observation suggests the intriguing possibility that these signalling systems collaborate to modulate genes necessary to elicit drug resistance. Understanding the molecular mechanism of endogenous glycopeptide resistance arising from this dual signalling synergy is being vigorous pursued.

The *yjbH*(K23stop) mutation displays both positive and negative effects upon glycopeptide resistance. By itself, the *yjbH*(K23stop) mutation displays little contribution to glycopeptide resistance, whereas when combined as a double mutant with either the *vraS*(G45R) or *stp1* null mutation, *yjbH*(K23stop) mutation reduces resistance. These findings suggest that mutation of *yjbH* exerts a negative influence on endogenous glycopeptide resistance achieved with either phosphosignalling pathway mutant alone. In contrast, when *yjbH* mutation is combined with the double mutant *stp1*(Q12stop)/*vraS*(G45R), a two-fold increase in modal teicoplanin MIC was observed (4 to 8 µg/ml) and was accompanied by increased colony counts indistinguishable from the parental triple mutant sequenced strain AR376. In this instance, *yjbH* clearly acts positively. The negative effects due to *yjbH*(K23stop) were also observed with vancomycin although its positive contribution to reduced susceptibility to this drug was less apparent in both spot PAP assays and modal MIC values. Future research will resolve in detail how YjbH and Spx contribute to glycopeptide resistance.

The drug resistance profiles of the three mutations detected in our study suggest that their temporal appearance was hierarchical. If either signalling mutation had occurred first, followed by *yjbH*, then the results we provide upon genetic reconstruction suggest little or no possibility for growth of the *yjbH*(K23stop)/*stp1* or *yjbH*(K23stop)/*vraS*(G45R) mutation on teicoplain 2 µg/ml and thus little chance for continued selection. In contrast, it is reasonable to assume that the dual signalling mutants emerged first, followed by the acquisition of the *yjbH* mutation. This genetic order has the effect of pushing the teicoplanin MIC from 4 µg/ml to 8 µg/ml. It is thus tempting to speculate that the effect of *yjbH*, either by itself, or more likely through Spx, is manifest only when a threshold pre-existing level of glycopeptide resistance is attained.

We have shown that loss of *yjbH* leads to stabilization of Spx protein levels and provide evidence that Spx modulates the expression of at least two genes (*msrA1* and *trfA*) involved in glycopeptide resistance. Spx is necessary for the upregulation of *msrA1*, encoding methionine sulfoxide reductase, an enzyme required to combat oxidative stress. *msrA1* (SA1257) is transcribed as a four-gene operon (SA1254-SA1257) and induced by several cell wall active antibiotics [24], [29], [30], [33]. In addition, activating oxidative stress defences is a plausible strategy to combat antibiotic stress since recent studies reveal that several antibiotics, including glycopeptides, kill cells by promoting the production of endogenous reactive oxygen species [28], [47], [48].

Teicoplanin resistance factor A, encoded by *trfA*, is also regulated by YjbH and Spx (A. Renzoni, manuscript in preparation). TrfA is important for glycopeptide resistance in several strain backgrounds including AR376 used in this and other studies, although its precise function has yet to be established [21]. Studies are underway to dissect which precise pathways are affected by the *trfA* mutation.

The three mutations uncovered in our study each contribute to enhanced cell wall thickness, although *vraS*(G45R) evokes only minimal change by itself and acts in conjunction with either of the other mutations. These findings could partly explain the reduced glycopeptide sensitivites observed in some, but not all of our strains, since in many instances no change in glycopeptide MICs were detected despite strong changes in cell wall thickness. To date, the majority of clinical glycopeptide resistant strains exhibit cell wall thickening [3], [11]; however, reduced susceptibility to glycopeptides can be achieved without alterations in cell wall thickness [49], [50]. Other metabolic changes must be affected by the mutations uncovered in our study which modulate low level glycopeptide resistance. While herein we present a detailed analysis suggesting an intricate interplay among signalling pathways in one particular strain, it is certainly possible that additional genes and pathways contributing to the acquisition of endogeneous glycopeptides resistance will be uncovered.

## Materials and Methods

### Bacterial strains and culture conditions

Bacterial strains used in this study are listed in Table 1. The NCTC8325 strain ISP794 (MIC = 1 µg/ml) and its teicoplanin-derivative AR376 (MIC = 8 µg/ml) are MLST type ST8 (3-3-1-1-4-4-3) and were described previously [21]. All *S. aureus* and *E. coli* strains were grown in Mueller-Hinton broth (MHB) and Luria-Bertani medium, respectively. When required, media were supplemented with the following antibiotics: 15 µg/ml chloramphenicol, 5 µg/ml erythromycin, 40 µg/ml kanamycin, and 3 µg/ml tetracycline.

### Genome sequencing

Genomic DNA (gDNA) was prepared from an overnight culture grown in MHB at 37°C as previously described [21]. Solexa technology was used to sequence the genomic DNA of both ISP794 and AR376 strains on an Illumina Genome Analyzer GAII (Illumina; Fasteris, SA, Geneva, Switzerland). The quality-controlled filtered reads, followed by alignment to the reference sequence NCTC8325 strain, were used to generate a consensus sequence for both ISP794 and AR376 strains, using the IUPAC ambiguity code. The generated consensus ISP794 and AR376 sequences were then compared to detect SNPs and InDel differences. All detected SNPs and InDels were subsequently verified or rejected by PCR of relevant genomic DNA and capillary sequencing. Additional details are available upon request.

### Construction and analysis of genetic point mutants

To test the contribution of each individual mutation to glycopeptide resistance, or reconstruct all possible combinations, we used genetic linkage analysis by conveniently tagging each mutation with a nearby resistance marker (Figure 1B), using pBT2 thermosensitive plasmid [21]. All plasmids were first electroporated into non-restricting strain RN4220 prior to electroporation into ISP794 and its derivative strains. After growth with applied marker selection at a non-permissive temperature for pBT2 replication, double cross-over events were screened using antibiotic markers and all linked mutants were confirmed by PCR and sequencing. We also verified in every case that the insertion of the antibiotic-resistant marker alone did not alter the susceptibility phenotype to either teicoplanin or vancomycin. The intergenic regions chosen were devoid of known small regulatory RNAs. While we cannot exclude that the markers used confer some changes to the cell which we do not detect, they are importantly neutral with respect to all assays used in this study to measure glycopeptide susceptibility. All antibiotic-linked SNP mutations were subsequently backcrossed to ISP794 using bacteriophage Φ80α-mediated generalized transduction. In some cases, allelic exchange using nearby markers linked to the wild type locus were used to remove one or several point mutations and restoration of wild type sequence. Non-cell wall active antibiotic selectable markers kanamycin, tetracycline, and erythromycin were obtained as previously described [21], [32]. Details of the construction of each single, pairwise, or reconstituted triple mutation are indicated below. In no case did we observe that single, double mutants, or the reconstructed triple mutant displayed altered growth rates or detectable fitness changes compared to ISP794.

### Generation of point mutant *vraS*(G45R) in ISP794

*VraS*(G45R) point mutation linked to a kanamycin-resistant marker was constructed in ISP794 as follows: a chromosomal fragment containing a nucleotide change in position 133 (C to T) of *vraS* open-reading frame (ORF) to generate an amino acid change from glycine to arginine, was amplified from ISP794 using primers described in Table S1. A restriction site (Bgl2) was used to ligate a kanamycin resistance marker at position 1972957 (NCTC 8325 genome sequence coordinates).

The resulting plasmid, pAR749, was electroporated into *vraS*::*ermB* mutant (described in [32] selecting for kanamycin resistance. Double cross-over events were screened on agar containing 40 µg/ml kanamycin and then replica streaked on 15 µg/ml chloramphenicol and 5 µg/ml erythromycin plates to screen for chloramphenicol and erythromycin-sensitive but kanamycin-resistant colonies. Clone AR774a was selected and used to backcross *vraS*(G45R) linked to kanamycin resistant marker to ISP794 using Φ80α lysates. A single mutant *vraS*(G45R) clone, AR774, was retained for further study.

### Generation of *stp1*(Q12stop) and *yjbH*(K23stop) single mutants in ISP794

A similar strategy was used to generate *stp1*(Q12stop) mutant in ISP794 strain. We first pre-marked strain AR376 by targeted insertion of a kanamycin resistance marker nearby the *stp1* gene (chromosomal locations 1142731 and 1142744 from NCTC 8325 genome sequence coordinates), using primer pairs described in Table S1. A restriction site (Bgl2) was used to insert a kanamycin resistant marker as described above. The resulting plasmid, pAR784, was electroporated into AR376, selecting with 40 µg/ml kanamycin and then replica streaked on 15 µg/ml chloramphenicol plates to screen for chloramphenicol-sensitive but kanamycin-resistant colonies. Clone AR802 was chosen and used to backcross the *stp1*(Q12stop) linked to kanamycin resistant marker to ISP794 using bacteriophage generalized transduction with Φ80α lysates. A single mutant *stp1*(Q12stop) AR826 was retained for further study.

To generate the *yjbH*(K23stop) mutant, strain AR376 was pre-marked by targeted insertion of an erythromycin resistance gene nearby *yjbH* gene in position 905525 (NCTC 8325 genome sequence coordinates) using plasmid pAR1063 generated with primers described in Table S1. One colony was selected and designated AR1077. Transfer of *yjbH*(K23stop) mutation into ISP794 strain was next performed by bacteriophage transduction using Φ80α lysates of AR1077 selecting for erythromycin-resistance marker. A single mutant *yjbH*(K23stop), AR1079, was fully verified by PCR and sequence analysis and retained for further study.

### Generation of pairwise and triple mutants

The double mutant *vraS*(G45R)-*stp1*(Q12stop) was constructed as follows: we first pre-marked strain AR376 nearby *stp1* gene (AR853) using plasmid pAR787 as described above, but instead inserting a tetracycline resistance marker at the same chromosomal location as we had inserted the kanamycin marker. Transfer of the *stp1*(Q12stop) mutation to *vraS*(G45R) mutant (AR774) was next performed by bacteriophage transduction using Φ80α lysates of AR853 selecting for the tetracycline-resistance marker. The selected kanamycin and tetracycline-resistant transductant (AR858) was analysed for acquisition of both *vraS*(G45R) and *stp1*(Q12stop) mutations by sequencing. The double mutant *vraS*(G45R)-*yjbH*(K23stop) was constructed as follows: we pre-marked strain ISP794 nearby *stp1* gene (AR860) using plasmid pAR784 as described above, but instead inserting a kanamycin resistance marker. Transfer of the *stp1* wild-type gene to AR376 was next performed by bacteriophage transduction using Φ80α lysates of AR860 selecting for the kanamycin-resistance marker. The selected kanamycin-resistant transductant (AR864) was analysed for acquisition of wild-type *stp1* gene by allelic exchange and the presence of both *vraS*(G45R) and *yjbH*(K23stop) mutations by sequencing. To construct the double mutant *stp1*(Q12stop)-*yjbH*(K23stop), we pre-marked strain ISP794 with a kanamycin resistance marker nearby wild-type *vraS* gene (AR758) using plasmid pAR712 as previously described [32]. Transfer of wild-type *vraS* gene to AR376 was next performed by bacteriophage transduction using Φ80α lysates of AR758 selecting for the kanamycin-resistance marker. The selected kanamycin-resistant transductant (AR854) was analysed for acquisition of wild-type *vraS* gene and the presence of both *stp1*(Q12stop) and *yjbH*(K23stop) mutations by sequencing.

Finally, to reconstruct the *vraS*(G45R)-*stp1*(Q12stop)-*yjbH*(K23stop) triple mutant, we transfered the *yjbH*(K23stop) mutation from AR376 by bacteriophage transduction using Φ80α lysates of AR1077 to the double mutant *vraS*(G45R)-*stp1*(K23stop) (AR858) selecting for the erythromycin-resistance marker. The selected erythromycin, kanamycin and tetracycline-resistant transductant (AR1082) was analysed for acquisition of *vraS*(G45R), *stp1*(Q12stop) and *yjbH*(K23stop) mutations by sequencing.

### Plasmids for complementation

Plasmid pMK4 was used to express the entire wild-type *vraR* operon under control of its own promoter and *stp1* or *yjbH* under the control of the heterologous *p*_HU_ or *p*_GlyS_ promoters (Table 1). Briefly, pMK4 plasmid containing Not1-Kpn *p*_GlyS_ or *p*_HU_ promoters were obtained as described [51], [52]. PCR fragments containing *vraR* operon, or promoterless *stp1* or *yjbH* were cloned into Kpn-Pst1 restriction sites using primers described in Table S1. All constructions were sequenced verified. Plasmids constructed in *E. coli* were first introduced into strain RN4220 prior to transformation of the corresponding mutant strains. Plasmids for negative controls in these experiments were either empty pMK4, or pMK4 harboring a heterologous gene, namely GFPuv4, driven by the same *p*_HU_ or *p*_GlyS_ promoters.

### Minimal inhibitory concentration (MIC) and population analysis

Broth macrodilution MIC assays were performed in triplicate as previously described according to CLSI (Clinical and Laboratory Standards Institute) standard procedures [21]. Results were reported as the modal MIC values. The spot PAP method was used to assess population analysis, as previously described [21], [32]. Bacterial cultures were adjusted to 0.5 MacFarland standard (1.5×10^8^ bacteria/ml), corresponding to an OD_600_ = 0.1. Serial log_10_ dilutions (resulting in a range 10^−1^ to 10^−5^) were prepared, then aliquots of each dilution (10 µl) were spotted on freshly prepared MH agar (MHA) containing different concentrations of teicoplanin or vancomycin. Viable colonies were examined after 48 h at 37°C. The results reported were consistant across at least five independent assays. It is important to emphasize that the MIC values reported throughout our studies were determined using broth macrodilution; this method may give higher values than other susceptibility testing methods. Indeed, a recent study conducted in our laboratory indicates that microdilution methods tend to underestimate glycopeptide resistance [53].

### Purification of recombinant Spx protein and antibody production

The open reading frame of the *spx* gene (SA0856 N315) was PCR amplified with primers indicated in Table S1 and cloned in pBluescriptII KS+. A sequenced verified *spx* fragment was next subcloned into *E. coli* expression vector pTYB12 (New England Biolabs) using Nde1-Pst1 sites, generating plasmid pAM1101. N-terminal intein-recombinant Spx protein was then purified using chitin affinity column (IMPACT system, New England Biolabs) as follows: *E. coli* strain ER2566 (New England Biolabs) containing pTYB12-Spx protein was grown in Luria-Bertani media containing carbenicillin at 100 µg/ml until an OD_600_ of 0.7, induced with 0.4 mM isopropyl–β-d-1-thiogalactopyranoside (IPTG) for an additional 2 h30 min at 22°C with vigorous shaking. Bacteria were harvested by low speed centrifugation, resuspended in buffer containing 0.1% Tween 20, followed by affinity chromatography and intein-mediated proteolytic cleavage of the affinity tag with 50 mM DTT. Recombiant Spx protein was eluted with 20 mM Tris-HCl pH 7.5, 500 mM NaCl and 1 mM EDTA, according to the manufacturer's recommendations. Thiol-induced intein cleavage resulted in the N-terminal attachment of three additional amino acids AlaGlyHis. Protein concentrations were determined using Bradford reagent (BioRad) and a bovine serum albumin standard. Recombinant Spx protein was stored at 4°C and used to generate polyclonal antibodies using the Rabbit Express™ extended protocol (Pickcell Laboratories, Netherlands).

### Western blot analysis

Total protein extracts were obtained as follows: 10 ml of an overnight culture of *S. aureus* strains in MHB were harvested. After centrifugation, cell pellets were washed and resuspended in 500 uL TES (10 mM Tris pH 7.5, 1 mM EDTA and 0.1% SDS). Bacterial cells were disrupted by adding 500 µl of acid washed glass beads (100–200 micron, Sigma) and using FastPrep cell disrupter (MP Biomedicals). The cell debris were separated from soluble protein extracts by centrifugation for 10 min at 4°C at 17000 rpm (JA-20 rotor Beckman) followed by a second centrifugation step to remove additional insoluble and aggregated proteins (30 min, 4°C, 17000 rpm). Supernatant was removed and proteins were concentrated on Amicon spin columns (4 kDa cut-off, Milian, Geneva, Switzerland). Protein concentrations were determined by Bradford assay (Bio-Rad). Aliquots of total proteins (50 µg) were loaded on 15% SDS-PAGE gels and blot transferred onto a polyvinylidene difluoride membranes (PVDF, Bio-Rad). After blocking using 5% (w/v) low fat milk in phosphate buffered saline, membranes were probed with a 1∶1500 dilution of Spx antibody followed by incubation with a secondary HRP-conjugated goat anti-rabbit antibody (1∶10,000 BioRad). Chemiluminescence was detected using the Western Pico Super Signal reagent (Pierce).

### Assays for emergence of teicoplanin resistance

Emergence of glycopeptide resistance was performed as previously described [21], [32]. Overnight bacterial cultures were adjusted to McFarland 2.0 in 0.9% NaCl and ca. 3×10^8^ CFU/ml were plated on Muller Hinton agar (BD BBL 211438) containing the indicated freshly prepared teicoplanin concentration and incubated for 48 h at 37°C. To determine the frequency of emergence, serial dilutions of each culture were also plated on MHB plates without drug. Results are expressed as the mean ± SEM of seven independent experiments for ISP794, AR774, AR826, AR1079 and five independent experiments for *spx*+ and Δ*spx*. A subset of colonies was retested by replica plating on selective agar plates to estimate the percentage of false positives (persisters cells which score as susceptible upon replating on selective medium) arising in each experiment. False positive rate of CFUs detected in the emergence assay were found to be 5–20% depending upon the experiment. The raw data are reported without correction and thus calculated emergence frequencies represent an upper limit. ISP794 used in this study is defective in *rsbU*. The effects of this mutation, its restoration, and the role of *sigB* in the emergence of teicoplanin resistance in this strain background have been thoroughly addressed in our previous study [32].

### Total RNA extraction

Overnight cultures of 8325-4*spx*-c and 8325-4Δ*spx* were diluted and grown at 37°C for 4 h in MHB without shaking. When indicated, sub-inhibitory concentrations of teicoplanin (0.5 µg/ml) were added and incubated for an additional hour. Bacteria were harvested and RNA extraction was performed as previously described [21]. The absence of contaminating DNA was verified by PCR using qRT-PCR probes in the absence of reverse transcription.

### Quantitative real-time qRT-PCR

mRNA levels were determined by quantitative RT-PCR (qRT-PCR) using the one-step reverse transcriptase qPCR Master Mix Kit (Eurogentec, Seraing, Belgium) as described [54]. Primers and probes were designed using PrimerExpress software (version 1.5; Applied Biosystems), and obtained from Eurogentec. *MsrA1* primers (from SA1257) and probe are: *msrA1*-334F 5′GGAAGTAACCTCTGGATCAAACGT, *MsrA*1-415R 5′CCCTACTTATGAACAGGTATGTACGAAT and *msrA1*-359T 5′ATTTGTACTGCTTCGACATGGCCGGTT. 16S primers and probe are described [54]. Reverse transcription and PCR were performed using primers and probes at a concentration of 0.2 and 0.1 µM, respectively. The *msrA1* mRNA levels were normalized on the basis of their 16S rRNA levels, which were assayed in each round of qRT-PCR as internal controls as described [54].

### Transmission electron microscopy

*S. aureus* strains were grown on TSB at 37°C under shaking conditions until post-exponential phase. One milliter of post-exponential phase bacteria was pelleted by low speed centrifugation, washed with PBS and fixed for 30 min in PBS containing 4% (v/v) glutaraldehyde. Fixed cells were further treated with 2% osmium tetroxide in buffer and immersed in a solution of 0,25% uranyl acetate to enhance membrane contrast. The pellets were dehydrated in increasing concentrations of ethanol followed by pure propylene oxide, then embedded in Epon resin. Thin sections for electron microscopy were stained with uranyl acetate and lead citrate and observed in a Technai 20 electron microscope (FEI Company, Eindhoven, The Netherlands). Digital images were captured at 37,000×. Cell wall thickness was determined using equatorial sections and four independent measurements for each image from random placement of a cardinal point grid. The reported values represent the mean of at least 40 independent images for each strain.

## Supporting Information

Figure S1 **Complementation of either** ***yjbH*** **(K23stop),** ***stp1*** **(Q12stop) or** ***vraS*** **(G45R) mutants by multicopy plasmid carrying wild-type genes.** (A) Spot plating population analysis of each single mutant and its corresponding complemented strain on teicoplanin. V* and s* complementation was tested on teicoplanin containing 0.5 µg/ml. Spot serial dilutions are indicated at the right margin. As *yjbH*(23stop) mutation has a subtle effect on teicoplanin resistance, complementation of the *yjbH* mutation was performed by introducing a multicopy plasmid carrying wild-type *yjbH* gene into the triple strain mutant, a condition where its role could be unambiguously assessed. Y* complementation was tested on teicoplanin containing 2 µg/ml. For convenience genes marked with an asterisk such as v*, s* and y* correspond to *vraS*(G45R), *stp1*(Q12stop) or *yjbH*(K23stop) mutations, respectively.
(TIF)

Table S1 **Primers used in this study.**
(DOC)
