# Supplementary figures and images for: Whole Genome Sequencing and Complete Genetic Analysis Reveals Novel Pathways to Glycopeptide Resistance in Staphylococcus aureus
Source: PLoS One. 2011 Jun 27;6(6):e21577. doi: 10.1371/journal.pone.0021577 (PMC3124529; doi:10.1371/journal.pone.0021577)

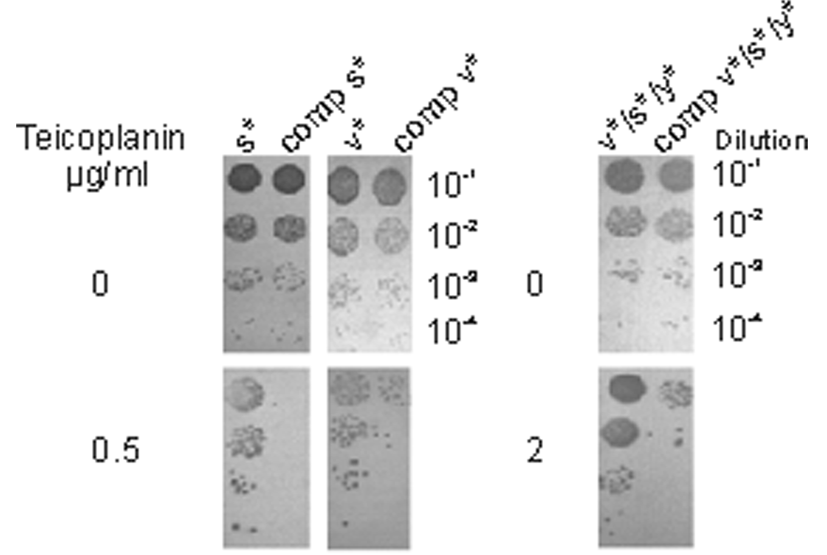

Supplement: Figure S1 — Complementation of either yjbH (K23stop), stp1 (Q12stop) or vraS (G45R) mutants by multicopy plasmid carrying wild-type genes. (A) Spot plating population analysis of each single mutant and its corresponding complemented strain on teicoplanin. V* and s* complementation was tested on teicoplanin containing 0.5 µg/ml. Spot serial dilutions are indicated at the right margin. As yjbH(23stop) mutation has a subtle effect on teicoplanin resistance, complementation of the yjbH mutation was performed by introducing a multicopy plasmid carrying wild-type yjbH gene into the triple strain mutant, a condition where its role could be unambiguously assessed. Y* complementation was tested on teicoplanin containing 2 µg/ml. For convenience genes marked with an asterisk such as v*, s* and y* correspond to vraS(G45R), stp1(Q12stop) or yjbH(K23stop) mutations, respectively. (TIF) [file pone.0021577.s001.tif]
