# Supplementary material for: Whole Genome Sequencing and Complete Genetic Analysis Reveals Novel Pathways to Glycopeptide Resistance in Staphylococcus aureus
Source: PLoS One. 2011 Jun 27;6(6):e21577. doi: 10.1371/journal.pone.0021577 (PMC3124529; doi:10.1371/journal.pone.0021577)
Supplement: Supplementary file 2 [file pone.0021577.s002.doc]

TABLE S1. Primers used in this study

Gene/Mutant/Protein	Primer	Primer sequence (5'-3')
vraS (G45R) mutant	VraSG45R PCRtop	AAAAGACTGGTATTCTGAATATTTGTGTATA
	VraSG45R PCRbot	TATACACAAATATTCAGAATACCAGTCTTTT
	SA1699EcoPst	CGGAATTCCTGCAGATGTCGAAAAAGGATCACTCTTCTTCAAAATACC
	12A Bgl2	GAAGATCTCGTAAGTAACTTTTCTTAATTCGATACG
	12B Bgl2	GAAGATCTCCAATCACAATATAACATCAAATAGACAC
	SA1703KpnBam	GGGGTACCGGATCCATGAACTATGTTGAACGTTATATTGAACAG
stp1 (Q12stop) mutant	Kpn1189F	GGGGTACCGTCACACAAGCATTGAAAGGT
	Inter1190BamH1R	CGGGATCCCAATAATAAATCACGCCATAATC
	BamH11190 inter F	CGGGATCCGGTCCTAGTACATTGATTTATG
	1192PstR	AACTGCAGCCACTTAGTGTAGTCATAAACG
yjbH (K23stop) mutant	Kpn0853	CGGGGTACCGTGCTGATATTGTAAGTATTTTAG
	0854BamH1R	CGCGGATCCCTAGTGCCTCTTTTATTATTTAAC
	BamH10855	CGCGGATCCAATTCTAGTACCTCTTTATTTTATC
	0855Pst1R	AAAACTGCAGCGTATATTCTAACACATCTTACA
Spx recombinant protein	Spx Kpn Nde F	GGGGTACCAAGGAGGAACACATATGGTAACATTATTTACTTCACC
	Spx Pst R	AAAACTGCAGTTAGTCAACCATACGTTGTGCTTC
pMK4 *p*HU *stp1*	Kpn-rbs-Stp1	CGGGTACCAAGGAGGAACAATCATGCTAGAGGCACAATTTTTTAC
	Stp1-Pst1	AAAACTGCAGTCATACTTTATCACCTTCAATAG
pMK4 *p*GlyS *yjbH*	Kpn0860	CGGGGTACCAAGGAGGAACAATCATGGAAAATAAGAGTCGTGAAG
	0860Pst1	AAAACTGCAGTTATTTTGATTTGATTTTAGGCAT

Underlined regions represent restriction enzymes sequences
